# *TEMPRANILLO* homologs in apple regulate flowering time in the woodland strawberry *Fragaria vesca*

**DOI:** 10.1038/s41598-023-29059-0

**Published:** 2023-02-03

**Authors:** Ata Dejahang, Naeimeh Maghsoudi, Amir Mousavi, Nader Farsad-Akhtar, Luis Matias-Hernandez, Soraya Pelaz, Kevin Folta, Nasser Mahna

**Affiliations:** 1 Department of Horticultural Sciences, Faculty of Agriculture, University of Tabriz, Tabriz, Iran; 2 Department of Plant Biotechnology, National Institute of Genetic Engineering and Biotechnology (NIGEB), Tehran, Iran; 3 Department of Plant Biology, Faculty of Natural Sciences, University of Tabriz, Tabriz, Iran; 4 Centre for Research in Agricultural Genomics (CRAG), CSIC-IRTA-UAB-UB, Campus UAB, Bellaterra (Cerdanyola del Valles), 08193 Barcelona, Spain; 5 Biotech Tricopharming Research, 08018 Barcelona, Spain; 6 Institució Catalana de Recerca i Estudis Avançats (ICREA), 08010 Barcelona, Spain; 7 Horticultural Sciences Department, University of Florida, Gainesville, FL 32611 USA

**Keywords:** Biotechnology, Genetics, Molecular biology, Plant sciences

## Abstract

The long juvenile period of fruit trees makes their breeding costly and time-consuming. Therefore, flowering time engineering and shortening the juvenile phase have become a breeding priority for the genetic improvement of fruit tree crops. Many economically valuable fruit trees belong to the *Rosaceae* family including apples and strawberries. *TEMPRANILLO* (*TEM*) acts as a key player in flowering time control through inhibiting *FT* function. Two genes with high sequence similarity with the *Arabidopsis TEM* genes were isolated from apple (*Malus domestica*). Due to the complexity of carrying out functional studies in apple, we characterized their function in woodland strawberry as well as their expression in apple. The expression of *MdTEM* genes in apple tissues from juvenile plants was dramatically higher than that in the tissues from adult trees. In woodland strawberry, the overexpression of *MdTEM* genes down-regulated *FvFT1*, *FvGA3OX1*, and *FvGA3OX2* genes in strawberry. The *MdTEM*-overexpressing lines exhibited delayed flowering, in terms of days to flowering and the number of leaves at flowering. While, *RNAi-mediated* silencing of *TEM* resulted in five days earlier flowering, with a lower number of leaves, a higher trichome density, and in some cases, caused in vitro flowering. According to these results and in silico analyses, it can be concluded that *MdTEM1* and *MdTEM2* can be considered as orthologs of *FvTEM* and probably *AtTEM* genes, which play an important role in regulating the juvenile phase and flowering time through regulating *FT* and GA biosynthetic pathway.

## Introduction

Many economically valuable fruit and ornamental crops belong to the *Rosaceae* family, and while there has been significant progress in the accumulation of genomics-level data in the last decade, the formidable task of linking genes to horticulturally relevant traits remains incomplete^1^. The plant’s life cycle is divided into juvenile and adult phases. Plants in the juvenile phase are incapable of response to floral signals even under inductive conditions^2,3^. Transition from juvenile to adult phase takes a long time in most of the fruit trees (e.g. 5–12 years in apples), which limits and slows breeding efforts such as backcrosses, inbreeding, or production of new hybrids^4^. Therefore, flowering time regulation is of key importance for breeding programs^5^. In the past decades, great progress has been obtained in understanding the molecular basis involved in flowering time regulation, especially in *Arabidopsis*^6^. Several genetic pathways which control flowering, including the vernalization, photoperiod, gibberellins, autonomous, age and ambient temperature pathways, have been characterized. These signaling pathways integrate developmental and environmental factors associated with the activation of a key floral regulator, *FLOWERING LOCUS T* (*FT*)^7,8^. FT protein is produced in leaves and moves through the phloem to the apex where it forms a complex with a bZIP transcription factor FD and activates the expression of the floral meristem identity genes to promote flowering^9^. The fact that plants are unable to initiate flowering during juvenility phase even in inductive environmental conditions proposes that inhibitory mechanisms may suppress FT expression during juvenility and prevent flowering^9,10^. Many plants require a given day length sometimes in combination with a certain temperature to initiate flowers. Flowering at right time associated with the seasonal and endogenous signals is vital for successful reproduction in plants^6^. Numerous genes influencing floral induction have been characterized. The orthologs of flowering-related genes of *Arabidopsis thaliana* (L.) have been isolated from Rosaceous crops like apple (*Malus domestica*), including *LEAFY (LFY)*, *APETELA1 (AP1)*, *AGAMOUS (AG)*, *TERMINAL FLOWER (TFL1)*, *BpMADS4*, and *SERRATED LEAVES AND EARLY FLOWERING (SEF)*^11–16^. *TEMPRANILLO* genes (*TEM1* and *TEM2*) belong to the plant-specific transcription factor RAV (related to ABI3/VP1) subfamily, which contain two DNA-binding domains, an AP2/ERF and a B3 DNA-binding domain^17,18^. *TEM* genes play a pivotal role in Arabidopsis flowering time. They directly repress *FT* transcription through binding to two regions in the *FT* gene 5ʹ untranslated region^17^ and also repress the GA biosynthetic genes *GA3OX1* and *GA3OX2* through binding to a sequence in the first exon^18,19^. Osnato et al.^19^ reported that *TEM* genes control floral transition through linking the photoperiod and GA-dependent flowering pathways to the regulation of the floral integrators. Double mutant *Arabidopsis* plants with reduced *TEM1* and *TEM2* activity flower earlier than the single *tem1* and *tem2* mutants, which flower earlier than wild-type plants^17^.

Plants undergo the transition to adult phase before they become capable to respond to the floral inductive signals. Thus, in Arabidopsis, the juvenile-to-adult transition is correlated with various morphological changes, including the formation of trichomes on the abaxial side of leaves^20^. Plant trichomes are specific epidermal protrusions which have several characteristics that vary between plant species and organs. The timing of abaxial trichome formation is associated with flowering time, consistent with the fact that the juvenile-to-adult vegetative phase change contributes to the acquisition of the competence to flower^21^. Studies revealed that *TEM* genes inhibit trichome initiation from the mesophyll, the lower layer of epidermis^22,23^. Fluorescently labeled GA3 exclusively accumulated in the mesophyll of cells, but not in the epidermis, suggesting that TEM plays an essential role in GA biosynthesis and distribution in the mesophyll, resulting in the epidermal trichome formation in Arabidopsis^23^.

*Fragaria vesca* offers several features that makes it an appropriate plant for functional genomics research in the Rosaceae family. It has a small diploid genome that is fully sequenced. The plant is small, self-compatible, easily transformed, has a small size, and may be propagated by runners and branch crowns as well as by seed^24–26^. *F. vesca* has both seasonal (SD) and perpetual (ever-bearing) flowering (LD) accessions with different photoperiodic responses^27,28^. In perpetual flowering accessions, LD advances flower induction, but plants eventually flower also under SD conditions^29–31^. Recently, the full length cDNA of *FvTEM* was isolated and characterized in silico, which revealed that it was 1152 bp in length predicted to encode 383 amino acids and homologous to *AtTEM*^32^. In the present study, the expression of the *MdTEM1* and *MdTEM2* genes and their downstream genes were analyzed in different tissues and growth phases in apple. Transgenic strawberry plants overexpressing *MdTEM1* and *MdTEM2* genes and those carrying *MdTEM1* RNAi constructs downregulating endogenous *FvTEM,* were generated and evaluated for their flowering time and trichome density.

## Materials and methods

### Plant material and growth conditions

Ten-year-old apple (*Malus domestica* cv. Golden Delicious) trees (Khalat-Poushan Reasearch Station, University of Tabriz, Tabriz, Iran) were used for DNA and RNA extractions, gene isolation and gene expression analyses. The seeds from perpetual flowering LD accession Hawaii-4 (H4, PI551572) of the woodland strawberry, *Fragaria vesca* L. ssp. *vesca f. alba* (Ehrh.) Staudt were used. This *Fragaria vesca* accession is available commercially (https://strawberryseedstore.com/store/Fragaria-vesca-ssp-vesca-Hawaii-4-p140266115) as well as from germplasm repositories (https://npgsweb.ars-grin.gov/gringlobal/accessiondetail?id=1446544). Experimental research and field studies on plants, including the collection of plant material, complied with relevant institutional, national, and international guidelines and legislation. The seeds were sterilized for 5 min in 70% (v/v) ethanol and in 1% sodium hypochlorite with 2 drops of Tween 20^®^ for 5 min and then rinsed in sterile distilled water several times before germination in Petri dishes containing ^1^/_2_ MS at pH 5.7 with 3% (w/v) sucrose. Seeds were cultured initially at 22 °C in dark for one week, and then transferred to the growth chamber with flowering non-inductive conditions (25 °C and 12/12 h light/dark photoperiod). High pressure sodium (HPS) lamps at 140 μmol m^−2^ s^−1^ were used to supplement natural light in the phytotron. Seedlings with one or more true leaves were transferred into the jars containing MS medium to increase size. The seedlings were transferred into fresh medium 2 weeks before the transformation, always in the same chamber. Though all plants in the growth chamber and phytotron were kept under flowering non-inductive conditions at 25 °C and 12/12 h light/dark photoperiod, the flowering experiments (describe below in 2.6) were done under inductive conditions at 25 °C and 16/8 h light/dark photoperiod.

### *MdTEM* genes isolation and vector construction

Two *AtTEM1*/*AtTEM2* homologs from apple and one homolog from woodland strawberry were isolated through a RT-PCR reaction with appropriate primer pairs (Supplementary Table S1) using the cDNA synthesized from the RNA extracted from the leaf samples, designated as *MdTEM1* (GenBank accession number: ON045007), *MdTEM2* (GenBank accession number: ON045008), and *FvTEM* (GenBank accession number: ON107496). The *MdTEM1* and *MdTEM2* genes were cloned into *pAlligator2*^33^ independently under the control of double enhanced *CaMV35* promoter and *NOS* terminator for overexpression experiments. Thus, we obtained the constructs named 35S:*MdTEM1* and 35S:*MdTEM2*. A 137 bp fragment of *MdTEM1* was also cloned into *pHellsgate12*^34^ under the control of *CaMV35S* promoter and *octopine synthase* terminator with two introns (catalase-1 and PDK) designed to trigger RNAi-mediated gene silencing, and the resulting construct was named *RNAi-TEM*. In both vectors, the *nptII* gene was used as selectable marker under the control of *NOS* promoter and terminator. Vectors carrying overexpression and RNAi constructs were incorporated into *Agrobacterium tumefaciens* strains GV3101 through electroporation (GenePulser, BioRad, USA).

### Plant transformation and regeneration

For transformation and regeneration of woodland strawberry, young fully expanded leaflets were placed with their adaxial side up in a Petri dish and sliced across and/or along the secondary veins to produce multiple cuts. Leaf sections were co-cultivated with *Agrobacterium* harboring an overexpression or RNAi construct in the medium containing MS salts and vitamins, 2% sucrose, 3 mg/L BA, 0.2 mg/L IBA and 0.7% agar^35^. After 3 days of co-cultivation, explants were washed with liquid MS containing 500 mg/L cefotaxime and placed with their abaxial side up in the selection media containing 3 mg/L BA, 0.2 mg/L IBA, 25 mg/L kanamycin and 250 mg/mL cefotaxime. Explants were subcultured with two-week intervals for 60–90 days until shoots appeared. Transformation efficiency for each construct was calculated as the percentage of the number of explants which produced PCR-positive plants out of the total number of inoculated explants.

### PCR analysis of transgenic strawberry plants

DNA was isolated from leaves of transformed and untransformed plants using a modified CTAB method^36^. Quality and quantity of the extracted DNA were checked by agarose gel and NanoDrop1000 spectrophotometer (NanoDrop Technologies, Wilmington, DE, USA). The putative transgenic plants were screened for the presence of T-DNA by polymerase chain reaction (PCR) analysis using NOS terminator and *MdTEM1* and *MdTEM2* primers for overexpression and using *MdTEM1* and *NPTII* primers for RNAi silencing experiments. Primers used for plant transformation validation by PCR are listed in Supplementary Table S1. The PCR reaction was carried out using 100 ng of genomic DNA under the following thermal cycling condition: 94 °C, 30 s; 57 °C, 30 s, and 72 °C, 50 s for 32 cycles. A 5 μL aliquot of each PCR reaction was analyzed by 1% agarose gel electrophoresis.

### RNA extraction, cDNA synthesis, and real-time PCR

For RNA extraction, different tissues of 10-year-old apple adult trees grown in the Khalat-Poushan Research Station of the University of Tabriz in the Spring of 2014 at ZT 6 were used. For the tissues from the juvenile apple seedlings, sampling was done also from the roots and the youngest fully opened leaves of the seedlings grown in a growth chamber at 25 °C under 16/8 h light/dark photoperiod at ZT 6. For the strawberry plants, the youngest fully opened leaves of the plants growing in a chamber at 25 °C under 16/8 h light/dark photoperiod at ZT 8 were used. Total RNA was extracted using modified CTAB method^37^ and then treated with RNase-Free DNase (Fermentas, Germany) according to the manufacturer’s recommendations. The purity and concentration of total RNA were measured using a NanoDrop 1000 spectrophotometer (NanoDrop Technologies, Wilmington, DE, USA) and first strand cDNA synthesized from 500 ng total-RNA using MMLV reverse transcriptase and oligo dT. qRT-PCR reactions were performed in a final volume of 20 μL on the Corbett Rotor-Gene 6000 (Corbett LifeScience) using Power SYBR green master mix (Life Technologies). The PCR conditions were as follows: 95 °C for 5 min, followed by 40 cycles of 95 °C for 15 s and at 60 °C for 35 s. Melting-curve analysis was conducted to verify the specificity of each primer using a temperature ramp starting from 65 °C to reach 95 °C with fluorescence measured every 1 °C. All qRT-PCRs were run in three technical and two biological replicates.

Relative transcript levels of *MdTEM1*, *MdTEM2* and *MdFT* genes from apple as well as *FvTEM, FvFT, FvGA3OX1,* and *FvGA3OX2* genes from strawberry were calculated by the 2^∆Ct^ for apple genes and 2^−∆∆Ct^ and − 1/2^−∆∆Ct^ for up and down-regulated genes in woodland strawberry, respectively^38^. *MdActin* for apple genes and *FvMSI1* for woodland strawberry genes were used as internal reference genes*.* Primers used for qRT-PCR analyses are listed in the Supplementary Table S1.

### Flowering time analysis

The regenerated independent transgenic lines and WT strawberry plants were rooted, transferred into pots and acclimatized in a phytotron under non-inductive conditions at 25 °C and 12/12 h light/dark photoperiod. Soilless growing media consisted of fertilized peat moss supplemented with 25% (v/v) of vermiculite were used. Two weeks later, the acclimatized plants were transferred to flowering inductive conditions at 25 °C and 16/8 h light/dark photoperiod. Flowering was recorded as the date the first flower opened. Flowering time data were taken daily at ZT8 for WT and transgenic strawberry lines by counting the number of days to flower and the number of rosette leaves right before flowering.

### Trichome analysis

In strawberry, four independent overexpressed lines (35S::*MdTEM1*#1 and 35S::*MdTEM1*#2, 35S::*MdTEM2*#1 and 35S::*MdTEM2*#2), and three independent silenced lines (RNAi-TEM #1, #2 and #3) as well as the WT plants with three biological replicates were studied. The fully expanded leaves from each line were placed in glass flasks containing 50 mL of 70% ethanol. Paradermal sections were collected from the central region of the abaxial surface of these adult leaves. The sections were washed using sterile distilled water for 3 min and immersed in 10% sodium hypochlorite solution until total clearing. The sections were then washed in distilled water and stained with 1% safranin for 3 h and rinsed in distilled water to remove excess dye. For each line, a total of 3 slides containing 5 sections each were prepared, making a total of 15 sections per line. The non-glandular trichomes were scored using a stereomicroscope equipped with a 14× objective lens^39^.

### Alignment, phylogenetic, and syntenic analyses

Multiple sequence alignment was performed using the deduced amino acid sequences of *MdTEM1*, *MdTEM2* and *FvTEM* with other *RAVI* orthologs from different plants. The sequences were aligned using the CLUSTALW alignment tool in MEGA11^40^. The evolutionary history was inferred using the Neighbor-Joining method^41^ and the optimal tree was shown. The percentage of replicate trees in which the associated taxa clustered together in the bootstrap test (500 replicates) were shown next to the branches^42^. The evolutionary distances were computed using the Poisson correction method^43^ and are in the units of the number of amino acid substitutions per site. This analysis involved 28 amino acid sequences. All ambiguous positions were removed for each sequence pair (pairwise deletion option). There were a total of 490 positions in the final dataset. Evolutionary analyses were conducted in MEGA11^40^. Amino acid sequence alignment of TEM proteins from apple, strawberry and Arabidopsis was carried out using CLUSTAL W method in MEGA11 and illustrated by CLC Genomics Workbench v21.0.5 (QIAGEN). Also, the genomic synteny was analyzed using SyMAP software v5.3.0^44,45^ and the graphs were obtained through the same package.

### Statistical analysis

ANOVA was conducted on the averages using the general linear model, and differences between means were analyzed by LSD test. All statistical analyses were conducted using the SPSS software package version 16.0 (SPSS, Inc., Chicago, Illinois). All graphs were drawn using MS Excel 2019. Image processing was done using GNU Image Manipulation Program (GIMP) wherever needed.

## Results

### MdTEM1, MdTEM2 and FvTEM are homologous to AtRAV proteins

**Figure 1:**
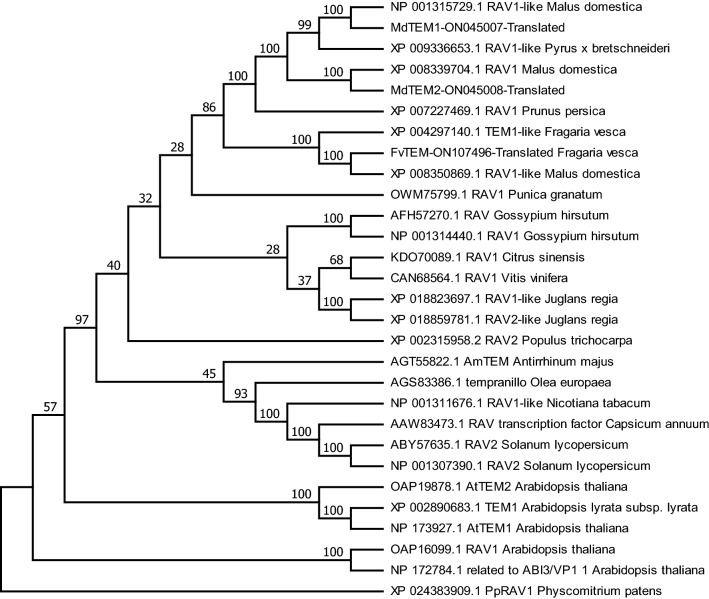
Phylogenetic analysis of *FvTEM* deduced amino acid sequences and other RAV sub-family class I members. The evolutionary history was inferred using the Neighbor-Joining method^1^. The optimal tree is shown. The percentage of replicate trees in which the associated taxa clustered together in the bootstrap test (500 replicates) are shown next to the branches^2^. The evolutionary distances were computed using the Poisson correction method^3^ and are in the units of the number of amino acid substitutions per site. This analysis involved 28 amino acid sequences. All ambiguous positions were removed for each sequence pair (pairwise deletion option). There were a total of 490 positions in the final dataset. PpRAV1 used as an outgroup for rooting. Evolutionary analyses were conducted in MEGA11^4^. Accession numbers are given next to the species name.

**Figure 2:**
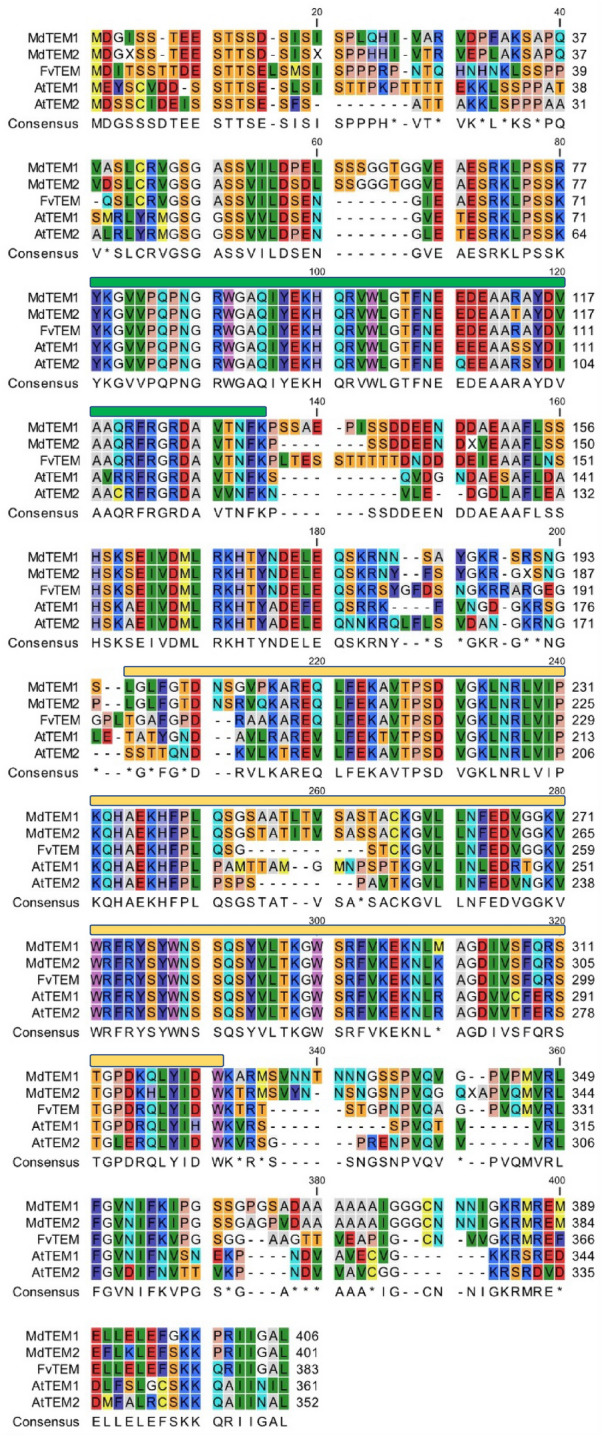
Amino acid sequence alignment of TEM proteins from apple, strawberry and Arabidopsis using CLUSTAL W method. The MdTEM1 (ON045007-translated), MdTEM2 (ON045008-translated), FvTEM (ON107496-translated), AtTEM1 (NP_173927.1) and AtTEM2 (NP_564947.1) sequences were used. The AP2 and B3 conserved domains were visualized by green and yellow bars, respectively. Alignment was carried out by MEGA11 and illustrated by CLC Genomics Workbench Version 21.0.5 (QIAGEN).

**Figure 3:**
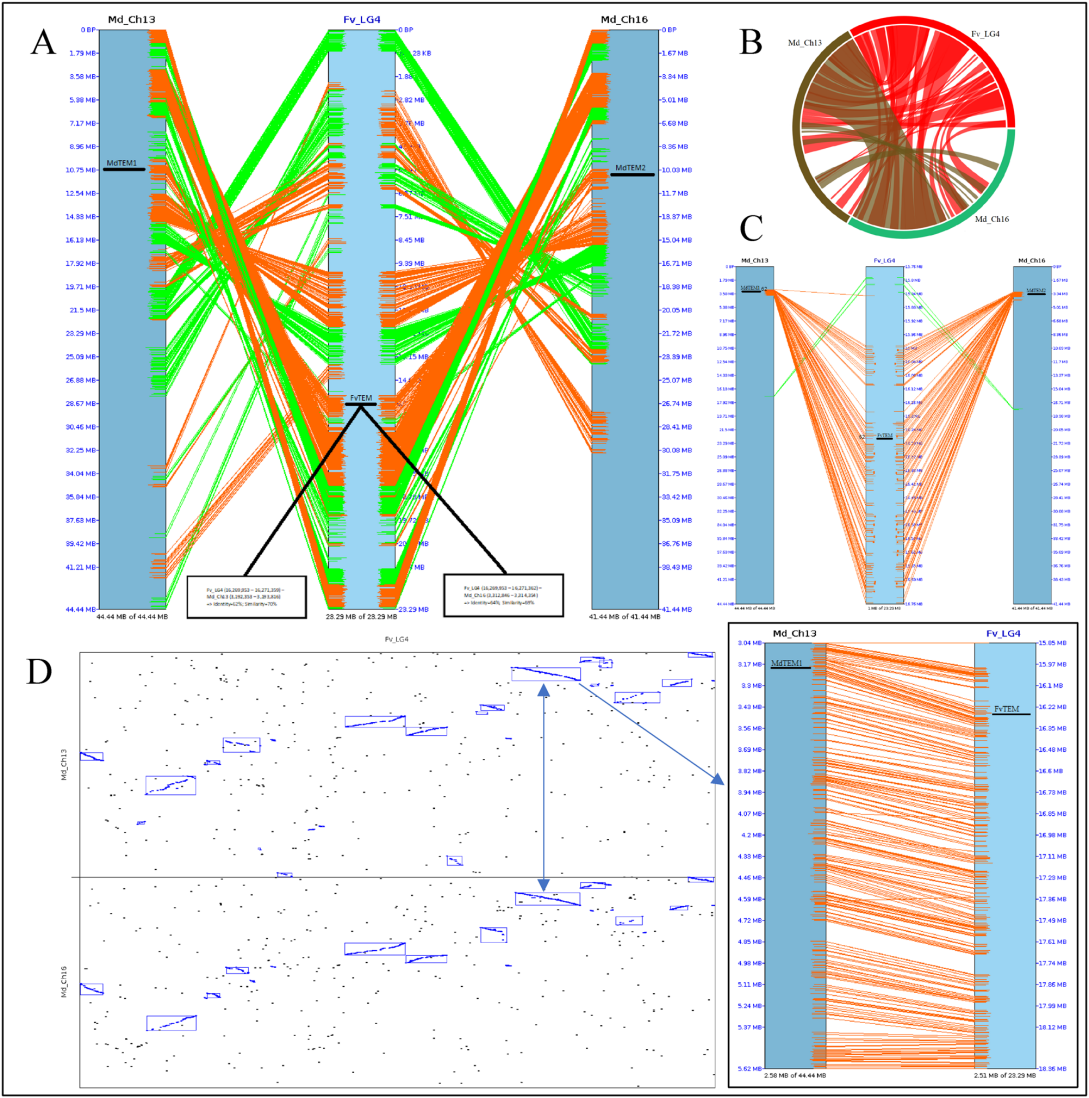
Syntenic analysis of genomic sequences of the linkage group 4 (Fv_LG4) of *F. vesca*, where *FvTEM* is located and chromosomes 13 of *M. domestica* (Md_Ch13) (where *MdTEM1* is positioned), and chromosome 16 of *M. domestica* (Md_Ch16) (where *MdTEM2* is located): (**A**) 2-D illustration; (**B**) circular illustration. (**C**) Focused illustration of the synteny between 1.0 Mb flanking sequences of *TEM* genes. (**D**) Dot-plot illustration of the syntenic regions (with more than 60% identity) pointing at the box containing the synteny blocks where *TEM* genes are positioned and focusing on the 2.5 Mb flanking sequences of *FvTEM* and *MdTEM* genes. The synteny has been analyzed using SyMAP software v5.3.0 and the graphs have been obtained through the same package.

The full-length cDNA of *MdTEM1*, *MdTEM2* and *FvTEM* consisted of the coding sequences of 1221, 1206 and 1065 bp, respectively, were isolated, predicted to encode a protein with 406, 401 and 355 amino acids, respectively. They had no intron and consisted of the AP2 and B3 domains which characterize it as a member of the RAV1 protein family. To determine the evolutionary relationships among the RAV1 family proteins, phylogenetic analysis was conducted by the amino acid sequences using Neighbor–Joining method for generating the phylogenetic tree. Phylogenetic analysis demonstrated that MdTEM1, MdTEM2 and FvTEM are homologous to RAV1-like proteins from other plants (Fig. 1). To illustrate the homology of the MdTEM1, MdTEM2, and FvTEM to each other and to AtTEM1 and AtTEM2, a CLUSTAL W alignment has been shown in Fig. 2. In silico comparison of FvTEM, MdTEM1, MdTEM2*,* AtTEM1, and AtTEM2 showed that they shared a high homology, and all have AP2 and B3 conserved domains (Fig. 2). Also, syntenic analysis of genomic sequences of the linkage group 4 of *F. vesca*, where FvTEM is located and chromosomes 13 of *M. domestica* where MdTEM1 is positioned, and chromosome 16 of *M. domestica* where MdTEM2 is located was carried out and demonstrated in many ways such as 2-Dimentional and Circular illustrations (Fig. 3). All results showed a large homology among *TEM* genes from all the examined species and a great possibility of orthology between the *TEM* genes from apple and wild strawberry.

### *MdTEM1* and* MdFT* show opposite expression patterns in apple

**Figure 4:**
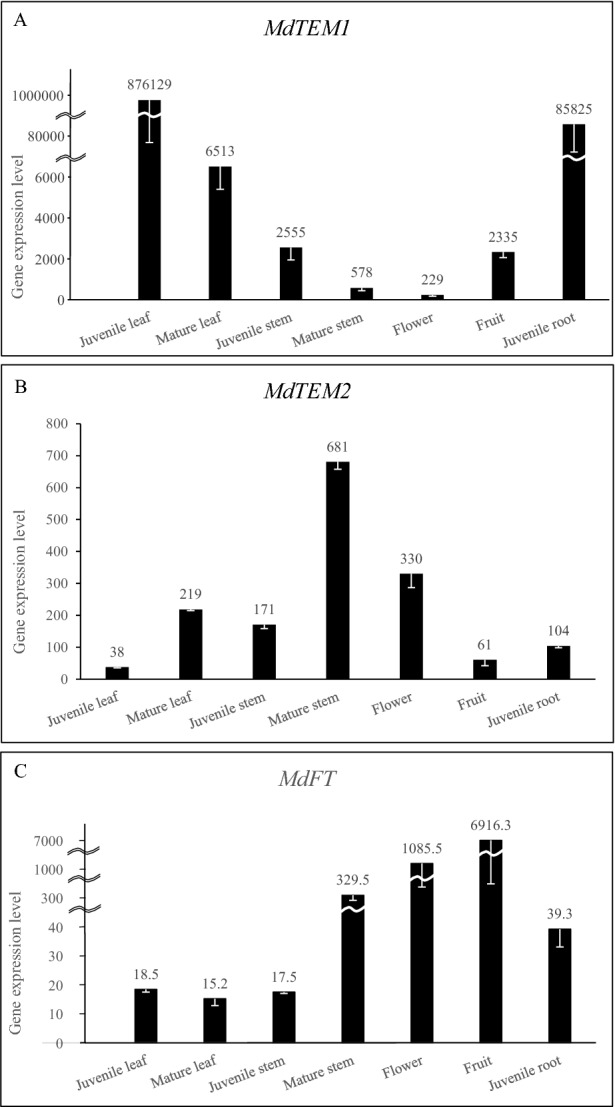
Expression levels of (**A**) *MdTEM1*, (**B**) *MdTEM2*, and (**C**) *MdFT* genes in different tissues in 10-year-old apple trees grown in the Khalat-Poushan Research Station of the University of Tabriz, Tabriz, Iran, at ZT 6 in the Spring of 2014 measured by qRT-PCR. The juvenile samples were taken from the seedlings grown in a growth chamber at 25 °C and 16/8 h light/dark photoperiod at ZT 6. Error bars indicate standard deviations for three technical and at least two biological replications.

The relative transcript levels of *MdTEM1*, *MdTEM2* and *MdFT* genes were measured in different tissues of apple by qRT-PCR. The highest expression levels of *MdTEM1* were observed in juvenile leaves and roots (Fig. 4), while the lowest transcript accumulation levels were obtained in flowers and mature stems. However, *MdTEM2* showed an almost opposite expression pattern, the highest expression levels were found in mature stems, flowers and fruits, whereas the lowest were observed in juvenile leaves and roots. The fact that two *MdTEM* genes have different expression pattern might suggest different specific roles in apple. On the other hand, *MdFT* had higher relatively expression in fruits, flowers and mature stems, opposite to *MdTEM1*. Based on other species information it may suggest a negative regulation of *MdFT* by *MdTEM1*.

### Generation of 35S::*MdTEM1*, 35S::*MdTEM2* and RNAi-*TEM* strawberry lines

Two overexpression (35S::*MdTEM1* and 35S::*MdTEM2*) and one RNAi silencing (RNAi-*TEM*) constructs were introduced into the diploid strawberry using *A. tumefaciens* GV3101. Putative transformants regenerated and rooted on MS medium containing kanamycin (Supplementary Fig. S1). The regenerated plantlets and non-transformed controls were then grown in pots in a phytotron at 25 °C under 16/8 h light/dark photoperiod (inductive conditions). Regenerated plants were screened by PCR using *MdTEM1* and *MdTEM2* and *NOS* terminator primers for both over-expression constructs and *MdTEM1* and *NPTII* primers for *RNAi* silencing construct. PCR analysis revealed the amplification of the expected specific fragments in transformed plants. No amplification was detected in the non-transgenic control (Supplementary Fig. S2). Transformation efficiency was calculated based on the percentage of inoculated explants that resulted in the production of PCR-positive plants. The results showed that the efficiency of transformation for *35S::MdTEM1*, *35S::MdTEM2* and RNAi-*TEM* constructs were 23.57, 17.07 and 32.5% respectively (Supplementary Table S2).

### Expression of *MdTEM* genes in strawberry affects the *F. vesca* flowering genes

**Figure 5:**
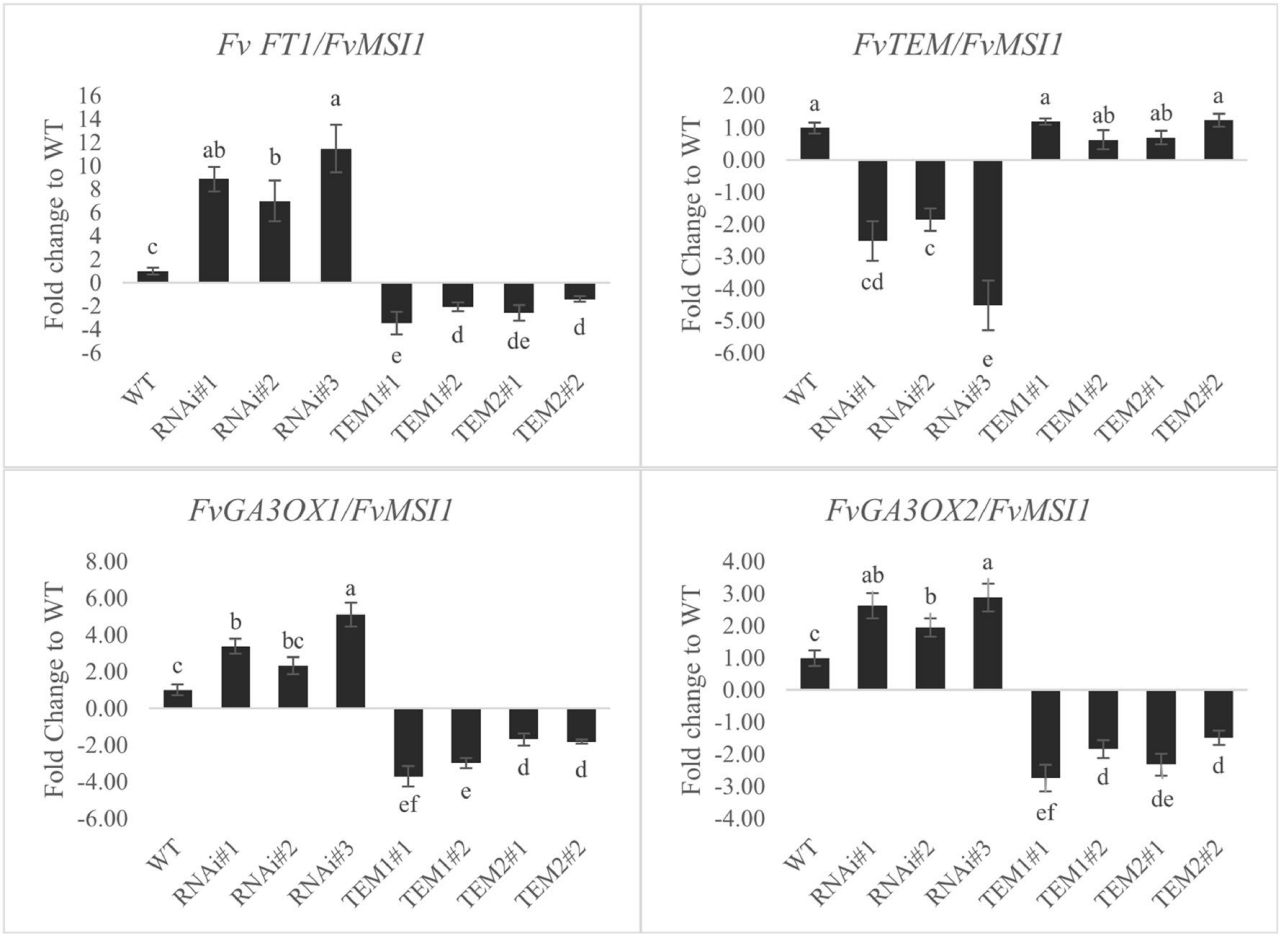
Relative expression of *FvFT1*, *FvTEM*, *FvGA3ox1* and *FvGA3ox2* genes in overexpressing and silencing H4 lines. The mean data are obtained from three biological and three technical replicates, all normalized to the expression level of *FvMSI1*. Samples were collected at ZT 8. Plants were raised in the growth chamber at 25 °C under 16/8 h light/dark photoperiod. Error bars indicate standard errors. Means were compared by LSD test and different letters show a significant difference at p ≤ 0.05.

**Figure 6:**
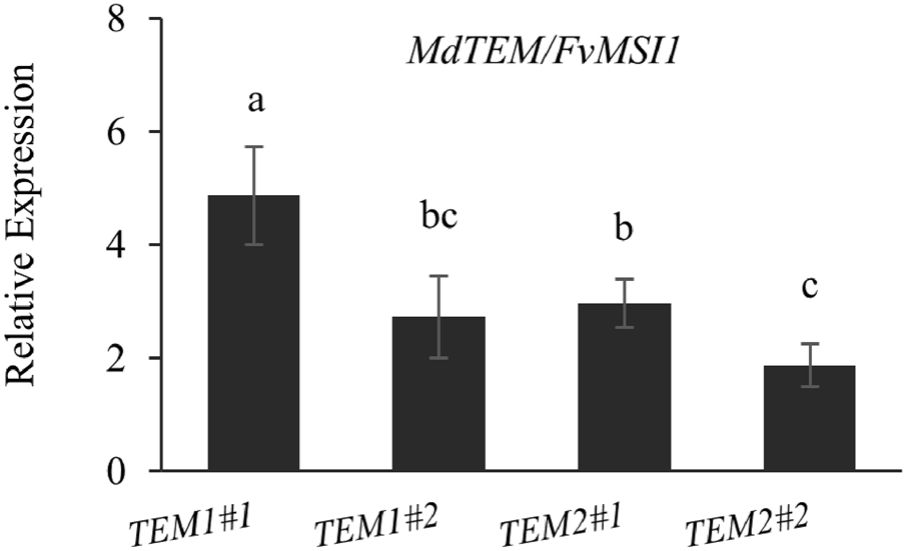
Relative expression of *MdTEM1* and *MdTEM2* genes in overexpressing *35S::MdTEM1* and *35S::MdTEM2* lines. RNA was measured using NanoDrop and 1 µg of RNA was used for each sample. Samples were collected at ZT 8 from fully expanded young leaves of plants in the growth chamber at 25 °C under 16/8 h. light/dark photoperiod. Error bars indicate standard errors. Means were compared by LSD test and different letters show a significant difference at p ≤ 0.05.

To study the role of *TEMPRANILLO* as a flowering-related transcription factor, the expression of *FvFT1*, *FvTEM*, *FvGA3OX1* and *FvGA3OX2* were measured by Real-Time quantitative PCR in the transgenic lines and non-transgenic control plants. qRT-PCR analysis showed that altered expression of *MdTEM* could affect the transcript levels of floral integration genes. Strikingly, RNAi-*TEM* could inhibit endogenous *FvTEM*, as RNAi-*TEM* lines exhibited lower *FvTEM* transcript levels. The RNAi-*TEM* lines showed significant increased transcript accumulation of *FvFT1*, *FvGA3OX1* and *FvGA3OX2* compared to control plants. Overexpression *MdTEM* lines exhibited a significant decrease in *FvFT1*, *FvGA3OX1* and *FvGA3OX2* transcript accumulation compared to WT plants (Fig. 5). However, the results also revealed that the *MdTEM1* and *MdTEM2* that were successfully expressed in 35S::*MdTEM1* and 35S::*MdTEM2* lines with different expression levels (Fig. 6), however, had no effect on endogenous *FvTEM* expression.

### *MdTEM* genes delay flowering time in strawberry

Flowering time was analyzed in the silenced and overexpressed lines compared with non-transgenic controls growing in chamber at 25 °C under 16/8 h inductive light/dark photoperiod. The number of leaves (for developmental stage) before flowering and the number of days (for chronological age) to flowering was assessed. The results indicated that the overexpression of *MdTEM1* and *MdTEM2* delayed flowering in *Fragaria vesca*, while the lower *FvTEM* activity produced in RNAi-*TEM* silencing lines significantly accelerated flowering (P < 0.01; Supplementary Table S3). Conversely, 25% of RNAi-*TEM* plants were flowered after 30 days when *MdTEM* overexpressing and control plants remained vegetative (Supplementary Fig. S3a,b). After 40 days, the percentage of 35S::*MdTEM1* and 35S::*MdTEM2* flowering plants were 36.4% and 37.5%, respectively, whereas this value for silencing RNAi-*TEM *and control plants were 100% (Table 1).

**Table 1:** Comparison of flowering rate in overexpressed and silenced lines after 30 and 40 days.

Construct	Plant number	Flowering rate (%)—30 days	Flowering rate (%)—40 days
35S::*MdTEM1‏*	11	0	36.4 (4)
35S::*MdTEM2*	8	0	37.5 (3)
*RNAi-tem*	12	25 (3)	100 (12)
WT	10	0	100 (10)

Numbers in parentheses represent the number of flowering plants.

**Figure 7:**
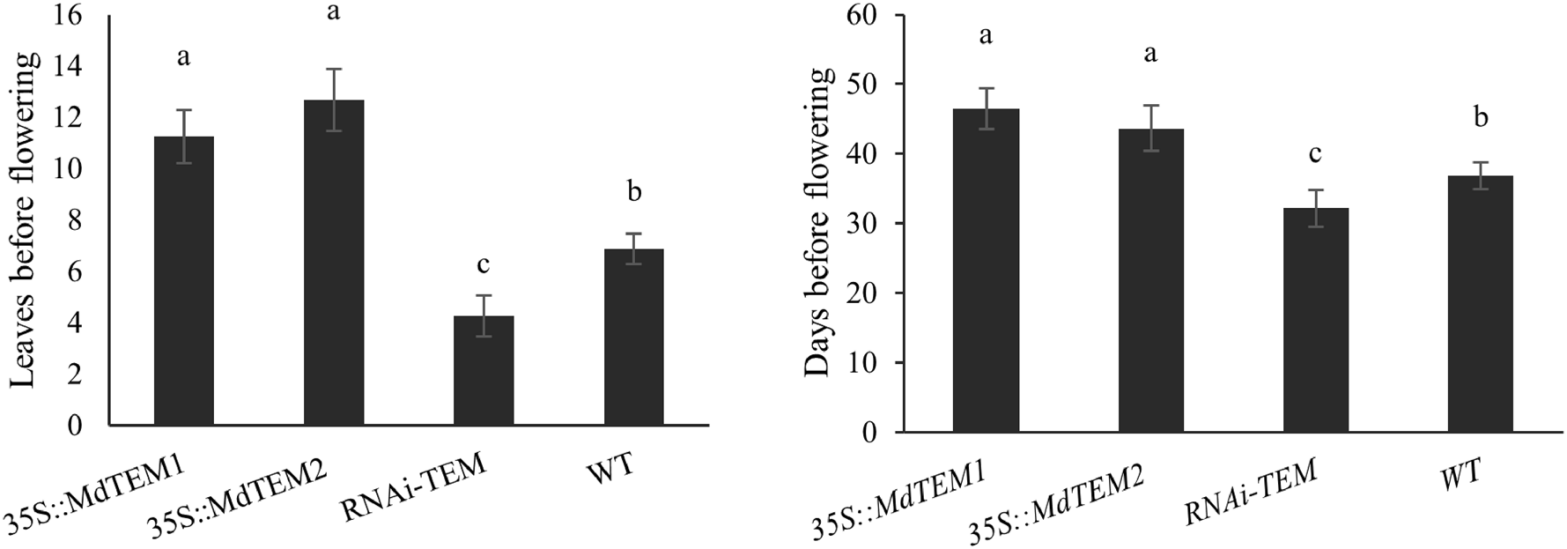
The mean number of leaves and number of days to flowering in *MdTEM1/2* overexpressed, silenced and wild type control plants of *Fragaria vesca* H4. Plants were raised at 25 °C under 16/8 h. light/dark photoperiod. Flowering was recorded as the date the first flower opened. Flowering time data were taken daily at ZT 8 for WT and transgenic lines. A number of 8–12 plants (replicates) were used to obtain each mean value. Error bars indicate the standard errors. Means were compared by LSD test and different letters show a significant difference at p ≤ 0.05.

In strawberry, overexpression of *MdTEM* caused delayed flowering, denoted as an increase in the number of leaves and the number of days upon flowering. The average number of leaves before flowering for RNAi-*TEM *and control plants were 4.25 and 6.88, respectively. While this value for the *35S::MdTEM1* and *35S::MdTEM2* lines were 11.25 and 12.67, respectively (Fig. 7). The average number of days before flowering for *35S::MdTEM1* and *35S::MdTEM2* were 46.5 and 43.67, respectively, while RNAi-*TEM* lines and control plants flowered after 32.17 and 36.88 days in inductive conditions, respectively. Our results showed that the RNAi-*TEM* silencing plants flowered with a significant smaller number of leaves and days before flowering compared to control plants. Strikingly, one of the RNAi-*TEM* lines flowered under in vitro non-inductive conditions (Supplementary Fig. S3c,d).

### Expression of MdTEM genes reduce trichome formation in *F. vesca*

**Figure 8:**
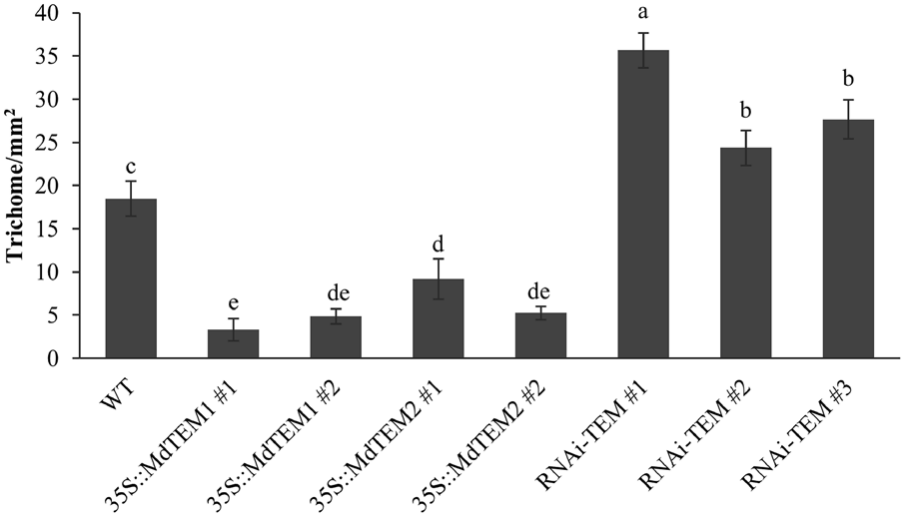
Number of trichomes per mm^2^ in different *MdTEM* overexpressed, silenced and wild type control plants of *Fragaria vesca*. For each line, a total of 3 slides containing 5 sections each were prepared, making a total of 15 sections per line with three biological replications. Error bars indicated standard errors. Means were compared by LSD test and different letters show a significant difference at p ≤ 0.05.

To evaluate the effect of *MdTEM* on the trichome formation in the diploid strawberry, the trichome distribution on the abaxial side of the four overexpressed, and three silenced lines as well as the wild-type strawberry plants were studied. The results showed that the expression level of *MdTEM* had a significant effect on the number of trichomes per mm^2^ (T mm^−2^) on the abaxial side of strawberry leaves. Number of trichomes per mm^2^ in RNAi-*TEM* and *35S::MdTEM* lines were higher and lower than those in the wild-type control plants, respectively (Fig. 8). The highest number of trichomes per mm^2^ (35.6) was belonged to the RNAi-*TEM* #1 line, while the 35S::*MdTEM1*#1 line produced the lowest trichome number (3.33 T mm^−2^). These differences were also observed in microscopic analysis of the abaxial side of their leaves (Supplementary Fig. S4). These data suggest that *MdTEM* and *FvTEM* genes also conserve the *AtTEM* function on negatively regulating trichome initiation.

## Discussion

Development of tree crops by breeding programs is very slow due to their long juvenile phase, which may take several years. One of the most important priorities of most breeding programs is to reduce the juvenile phase and accelerate the flowering process. Decreasing juvenility through the use of genetic engineering methods may hasten the production of new cultivars that are desperately needed to meet contemporary challenges, such as changes in climate and pest/pathogen threats. In this study, we produced early flowering lines by down-regulation of *FvTEM-like* genes through RNAi-mediated gene silencing in diploid strawberry, an appropriate model for apple and other species in the *Rosaceae* family. This strategy could be used in other species with a long juvenile phase. TEM1 and TEM2 belong to RAV transcription factor family, have been recognized as flowering repressor and juvenility regulator. Our previous study identified FvTEM as a RAV family member, which contained B3 and AP2 domains and can act as floral repressors^32^.

The synteny analysis here between the linkage group 4 of wild strawberry (Fv_LG4) and two chromosomes of apple (13 and 16) containing *TEM* genes demonstrated that there is a vast homology between these chromosomes including the regions that these genes are located. Based on the functional results and in silico examinations, it can be postulated that the *TEM* genes characterized here are orthologous–paralogous genes. Also, there was a huge synteny between the apple chromosomes 13 and 16 which can prove a vast genomic duplication in these apple chromosomes. In addition to all these, the synteny and conservation between Fv_LG4 and apple chromosomes 13 and 16, can be a clue for sharing a common ancestral origin.

In the present study, we produced early-flowering strawberry plants which flowered approximately 5 d before non-transformed control plants and even as early as in vitro flowering. Recently, early-flowering strawberry plants have also been produced using ALSV vector containing the *Arabidopsis thaliana FT* gene^46^ and using the *Eriobotrya japonica LEAFY* gene^6^. We have shown that the overexpression of *MdTEM1* and *MdTEM2* delayed flowering time in *Fragaria vesca*, indicating that these genes have similar functions to *AtTEM* genes. It has been described that FT protein is transported from leaf to the apex and activates downstream genes such as *SOC1*, *LFY*, and *AP1*, resulting in flower induction^9^. On the other hand, gibberellins (GAs) act not only to provoke the growth of plant organs, but also their accumulation upregulates *SOC1* and *LFY* and hence promotes phase transitions during development^19,47^. In the present research, reciprocal relationships between *TEM* with *FT* and *GA3OX1* and *GA3OX2* were observed, with increasing mRNA levels of *TEM* in overexpressed lines, the levels of *FT* and *GA3OX1* and *GA3OX2* are decreased and therefore flowering is delayed. Osnato et al.^19^ reported that constitutive overexpression of *TEM1* in *Arabidopsis* resulted in down-regulation of *GA3OX* genes by binding to their first exon, whereas *tem1-1* and *tem1–1 tem2–2* mutants showed an up-regulation in *GA3OX1* and *GA3OX2* expression. Similarly, down-regulation of *FvTEM* in RNAi-*TEM F. vesca* lines resulted in an increased level of *FvFT1* and *FvGA3OX1* and *FvGA3OX2* transcripts, leading to early flowering via both pathways, i.e. *FT* and GAs. We have also seen a positive correlation between the expression of *MdGA3OX1* and *MdSOC1* in apple leaves and flower buds (Unpublished data), which can be a clue for the functionality of GAs on flower induction in apple as well. All these results were in a good agreement with the previous studies that characterize *TEM* as a floral repressor^10,17,19,48^.

Perpetual flowering woodland strawberry *F. vesca* accessions (often called remontant or everbearing) such as Hawaii-4 (used in this research) have been considered as day-neutral or temperature-dependent LD plants in the previous studies^49–52^. In our research, in the background of the Hawaii-4, silencing *FvTEM* released the expression of *FvFT1* and it could evoke early flowering. The role of *FvFT1* in the LD flowering *F. vesca* was evaluated by Koskela et al.^30^ via RNAi-mediated silencing of *FvFT1* in the Hawaii-4 and it was found that all of these RNAi lines were clearly late flowering under LD. The same result obtained from the overexpression of *MdTEM* genes in our research, which in turn, downregulated *FvFT1*. These results suggest that in the LD-grown Hawaii-4, *FvTEM* is a suppressor of *FvFT1* and the latter is required for the normal upregulation of floral meristem identity genes, marking the beginning of floral initiation at the apex^53–55^.

Nevertheless, in seasonal flowering accessions of *F. vesca* (SD plants), a strong floral repressor, *FvTFL1*, has been shown to control seasonal flowering. In SD plants, the upregulation of *FvFT1* upregulates *FvTFL1* and the latter downregulates *FvFUL* and *FvAP1* and suppresses flowering^30,56^, whereas in perpetual flowering accessions, *FvTFL1* alleles are non-functional with a 2 base pair deletion in their first exon and upregulation of *FvFT1* can promote flowering^30^.

It can be concluded that *MdTEM* could recall the floral repressor function of *FvTEM,* and the expression level of *FvTEM* determines flowering time through *FvFT1* and GA biosynthetic genes expression in strawberry. The higher expression of *MdTEM* genes significantly delayed flowering process in woodland strawberry, which was in a good agreement with the results of Sgamma et al.^10^ which showed that a *TEM* ortholog from *Antirrhinum majus* (*AmTEM*) postponed the transition process from the juvenile to adult phase in Arabidopsis. The results obtained here on the expression profiles of *TEM* genes in apple and strawberry, functional outcomes in strawberry, and their proved homology between each other and among other *RVA1-like* genes including Arabidopsis *RAV1* genes via phylogenetical and syntenic in silico analyses all can be reasons to conclude that *TEM* genes in apple, strawberry and possibly Arabidopsis are functionally orthologous genes. The fact that two *MdTEM* genes have different expression patterns might suggest different specific roles, but possibly the result of a gene duplication in apple. However, it is likely that in strawberry, one gene is responsible for the role of both *TEM1* and *TEM2* genes. Accordingly, we observed in the woodland strawberry that *FvTEM* silencing affected both flowering time and trichome density, as described for both *tem1 tem2 Arabidopsis* mutants^19,23^.

Furthermore, the results revealed that the highest density of trichomes was observed on the abaxial side of RNAi-*TEM* transgenic strawberry lines which was higher than that in the control plants and much higher than that in the *35S::MdTEM* lines. These results are also in agreement with the fact that *TEM1* and *TEM2* not only suppress the floral induction but also inhibit the trichome formation through the GA biosynthesis pathway and the regulation of transcription factors that regulate trichome initiation^18,23^. In fact, flowering is not the only process controlled by RAV proteins, but has also been found to be involved in other plant growth processes such as trichome formation, leaf senescence, and responses to pathogenic infections and to abiotic stresses^23,57^. Moreover, it has been shown that the strawberry cultivars with a higher density of non-glandular trichomes have had significantly fewer western flower thrips (*Frankliniella occidentalis*) than the cultivars with lower densities of non-glandular trichomes on the upper leaf surface^58^. From the breeding perspective, manipulating the number of trichomes and increasing their density on the leaf surface which happened in RNAi-*TEM* transgenic strawberry lines in this research, can be considered an interesting approach to improve pest resistance in strawberries. In summary, downregulation of *FvTEM* expression seems a good strategy to generate two interesting traits at once, pest resistance and early flowering.

## Supplementary Information

Supplementary Information.

## Data Availability

All data generated or analyzed during this study are included in this published article (and its Supplementary Information files) or are available through the following accession numbers and links. The isolated genes’ CDS sequences were deposited at NCBI with the accession numbers: ON045007 (*MdTEM1*), ON045008 (*MdTEM2*), and ON107496 (*FvTEM*). Other genes and their NCBI accession numbers used in this research are: AGT55822.1 *Tempranillo* [*Antirrhinum majus*]; XP_002890683.1 TEM1 [*Arabidopsis lyrata* subsp. lyrata]; OAP16099.1 RAV1 [*Arabidopsis thaliana*]; NP_172784.1 related to ABI3/VP1 1 [*Arabidopsis thaliana*]; NP_173927.1 TEM1 [*Arabidopsis thaliana*]; NP_564947.1 related to ABI3/VP1 2 [*Arabidopsis thaliana*]; AAW83473.1 RAV transcription factor [*Capsicum annuum*]; KDO70089.1 RAV1 [*Citrus sinensis*]; XP_004297140.1 TEM1-like [*Fragaria vesca*]; AFH57270.1 RAV [*Gossypium hirsutum*]; NP_001314440.1 RAV1 [*Gossypium hirsutum*]; XP_018823697.1 RAV1-like [*Juglans regia*]; XP_018859781.1 RAV2-like [*Juglans regia*]; XP_008339704.1 RAV1 [*Malus domestica*]; NP_001315729.1 RAV1-like [*Malus domestica*]; XP_008350869.1 RAV1-like [*Malus domestica*]; NP_001311676.1 RAV1-like [*Nicotiana tabacum*]; AGS83386.1 Tempranillo [*Olea europaea*]; XP_002315958.2 RAV2 [*Populus trichocarpa*]; XP_007227469.1 RAV1 [*Prunus persica*]; OWM75799.1 RAV1 [*Punica granatum*]; XP_009336653.1 RAV1-like [*Pyrus* × *bretschneideri*]; ABY57635.1 RAV2 [*Solanum lycopersicum*]; NP_001307390.1 RAV2 [*Solanum lycopersicum*]; CAN68564.1 RAV1 [*Vitis vinifera*]; XM_004307240.2 *FvMSI1* [*Fragaria vesca*]; JN172098.1 *FvFT1* [*Fragaria vesca*]; XM_004302902.2 *FvGA3OX1* [*Fragaria vesca*]; XM_004288777.2 *FvGA3ox2* [*Fragaria vesca*]; DQ535887.1 *MdFT* [*Malus domestica*]; XM_008344381.3 *MdActin* [*Malus domestica*]; OAP19878.1 AtTEM2 [*Arabidopsis thaliana*]; XP_024383909.1 PpRAV1 [*Physcomitrium patens*]; tNOS NOS terminator and NPTII sequences are downloadable from the Snapgene website: (https://www.snapgene.com/local/fetch.php?set=plant_vectors&plasmid=pHELLSGATE_8).

## References

[1] Folta KM, Gardiner SE (2009). Genetics and Genomics of Rosaceae.

[2] Huijser P, Schmid M (2011). The control of developmental phase transitions in plants. Development.

[3] Yamagishi N, Kishigami R, Yoshikawa N (2014). Reduced generation time of apple seedlings to within a year by means of a plant virus vector: A new plant-breeding technique with no transmission of genetic modification to the next generation. Plant Biotechnol. J..

[4] Flachowsky H, Hanke MV, Peil A, Strauss S, Fladung M (2009). A review on transgenic approaches to accelerate breeding of woody plants. Plant Breed..

[5] Jung C (2017). Flowering time regulation: Agrochemical control of flowering. Nat. plants.

[6] Liu Y (2017). Over-expression of EjLFY-1 leads to an early flowering habit in strawberry (*Fragaria* × *ananassa*) and its asexual progeny. Front. Plant Sci..

[7] Fornara F, de Montaigu A, Coupland G (2010). SnapShot: Control of flowering in Arabidopsis. Cell.

[8] Wellmer F, Riechmann JL (2010). Gene networks controlling the initiation of flower development. Trends Genet..

[9] Corbesier L (2007). FT protein movement contributes to long-distance signaling in floral induction of Arabidopsis. Science.

[10] Sgamma T, Jackson A, Muleo R, Thomas B, Massiah A (2014). TEMPRANILLO is a regulator of juvenility in plants. Sci. Rep..

[11] Kotoda N (2002). Overexpression of MdMADS5, an APETALA1-like gene of apple, causes early flowering in transgenic Arabidopsis. Plant Sci..

[12] Esumi T, Tao R, Yonemori K (2005). Isolation of LEAFY and TERMINAL FLOWER 1 homologues from six fruit tree species in the subfamily Maloideae of the Rosaceae. Sex. Plant Reprod..

[13] Flachowsky H, Peil A, Sopanen T, Elo A, Hanke V (2007). Overexpression of BpMADS4 from silver birch (*Betula pendula* Roth.) induces early-flowering in apple (*Malus× domestica* Borkh.). Plant Breed..

[14] Kotoda N, Wada M (2005). MdTFL1, a TFL1-like gene of apple, retards the transition from the vegetative to reproductive phase in transgenic Arabidopsis. Plant Sci..

[15] Wada M, Cao Q-F, Kotoda N, Soejima J-I, Masuda T (2002). Apple has two orthologues of FLORICAULA/LEAFY involved in flowering. Plant Mol. Biol..

[16] Maghsoudi N, Mahna N, Sokhandan-Bashir N, Baghban-Kohnehrooz B (2015). Identification and isolation of a homolog of AtSEF gene from apple (*Malus domestica*). Int. J. Biosci..

[17] Castillejo C, Pelaz S (2008). The balance between CONSTANS and TEMPRANILLO activities determines *FT* expression to trigger flowering. Curr. Biol..

[18] Matías-Hernández L, Aguilar-Jaramillo AE, Marín-González E, Suárez-López P, Pelaz S (2014). RAV genes: Regulation of floral induction and beyond. Ann. Bot..

[19] Osnato M, Castillejo C, Matías-Hernández L, Pelaz S (2012). TEMPRANILLO genes link photoperiod and gibberellin pathways to control flowering in Arabidopsis. Nat. Commun..

[20] Wang L (2019). A spatiotemporally regulated transcriptional complex underlies heteroblastic development of leaf hairs in *Arabidopsis thaliana*. EMBO J..

[21] Aguilar-Jaramillo AE (2019). TEMPRANILLO is a direct repressor of the micro RNA miR172. Plant J..

[22] Jiao Y (2016). Trichome formation: Gibberellins on the move. Plant Physiol..

[23] Matías-Hernández L (2016). TEMPRANILLO reveals the mesophyll as crucial for epidermal trichome formation. Plant Physiol..

[24] Folta KM, Davis TM (2006). Strawberry genes and genomics. Crit. Rev. Plant Sci..

[25] Pantazis CJ (2013). Development of an efficient transformation method by *Agrobacterium tumefaciens* and high throughput spray assay to identify transgenic plants for woodland strawberry (*Fragaria vesca*) using NPTII selection. Plant Cell Rep..

[26] Slovin JP, Schmitt K, Folta KM (2009). An inbred line of the diploid strawberry *Fragaria vesca* F. semperflorens for genomic and molecular genetic studies in the Rosaceae. Plant Methods.

[27] Brown T, Wareing P (1965). The genetical control of the everbearing habit and three other characters in varieties of *Fragaria vesca*. Euphytica.

[28] Heide OM, Sønsteby A (2007). Interactions of temperature and photoperiod in the control of flowering of latitudinal and altitudinal populations of wild strawberry (*Fragaria vesca*). Physiol. Plant..

[29] Koskela EA (2017). Altered regulation of TERMINAL FLOWER 1 causes the unique vernalisation response in an arctic woodland strawberry accession. New Phytol..

[30] Koskela EA (2012). Mutation in TERMINAL FLOWER1 reverses the photoperiodic requirement for flowering in the wild strawberry *Fragaria vesca*. Plant Physiol..

[31] Rantanen M (2014). Light quality regulates flowering in FvFT1/FvTFL1 dependent manner in the woodland strawberry *Fragaria vesca*. Front. Plant Sci..

[32] Dejahang A, Mahna N, Akhtar NF, Mousavi A (2018). Identification, isolation and in silico characterization of *Fragaria vesca* homologue of *TEMPRANILLO* gene. Bionature.

[33] Bensmihen S (2004). Analysis of an activated ABI5 allele using a new selection method for transgenic Arabidopsis seeds. FEBS Lett..

[34] Helliwell C, Waterhouse P (2003). Constructs and methods for high-throughput gene silencing in plants. Methods.

[35] Oosumi T (2006). High-efficiency transformation of the diploid strawberry (*Fragaria vesca*) for functional genomics. Planta.

[36] Doyle JJ, Doyle JL (1990). Isolation ofplant DNA from fresh tissue. Focus.

[37] Bowtell D, Sambrook J (2002). DNA Microarrays: A Molecular Cloning Manual.

[38] Schmittgen TD, Livak KJ (2008). Analyzing real-time PCR data by the comparative C T method. Nat. Protoc..

[39] Figueiredo AST, Resende JTV, Morales RGF, Gonçalves APS, Da Silva PR (2013). The role of glandular and non-glandular trichomes in the negative interactions between strawberry cultivars and spider mite. Arthropod-Plant Interact..

[40] Tamura K, Stecher G, Kumar S (2021). MEGA11: Molecular evolutionary genetics analysis version 11. Mol. Biol. Evol..

[41] Saitou N, Nei M (1987). The neighbor-joining method: A new method for reconstructing phylogenetic trees. Mol. Biol. Evol..

[42] Felsenstein J (1985). Confidence limits on phylogenies: An approach using the bootstrap. Evolution.

[43] Zuckerkandl E, Pauling L (1965). Evolving Genes and Proteins.

[44] Soderlund C, Bomhoff M, Nelson WM (2011). SyMAP v3.4: A turnkey synteny system with application to plant genomes. Nucleic Acids Res..

[45] Soderlund C, Nelson W, Shoemaker A, Paterson A (2006). SyMAP: A system for discovering and viewing syntenic regions of FPC maps. Genome Res..

[46] Li C, Yamagishi N, Kasajima I, Yoshikawa N (2019). Virus-induced gene silencing and virus-induced flowering in strawberry (*Fragaria× ananassa*) using apple latent spherical virus vectors. Horticult. Res..

[47] Mutasa-Göttgens E, Hedden P (2009). Gibberellin as a factor in floral regulatory networks. J. Exp. Bot..

[48] Marín-González E (2015). Short vegetative phase up-regulates TEMPRANILLO2 floral repressor at low ambient temperatures. Plant Physiol..

[49] Sønsteby A, Heide O (2007). Long-day control of flowering in everbearing strawberries. J. Hortic. Sci. Biotechnol..

[50] Weebadde C (2008). Using a linkage mapping approach to identify QTL for day-neutrality in the octoploid strawberry. Plant Breed..

[51] Bradford E, Hancock JF, Warner RM (2010). Interactions of temperature and photoperiod determine expression of repeat flowering in strawberry. J. Am. Soc. Hortic. Sci..

[52] Stewart PJ, Folta KM (2010). A review of photoperiodic flowering research in strawberry (*Fragaria* spp.). Crit. Rev. Plant Sci..

[53] Mandel MA, Yanofsky MF (1995). A gene triggering flower formation in Arabidopsis. Nature.

[54] Ferrándiz C, Gu Q, Martienssen R, Yanofsky MF (2000). Redundant regulation of meristem identity and plant architecture by FRUITFULL, APETALA1 and CAULIFLOWER. Development.

[55] Mouhu K (2009). Identification of flowering genes in strawberry, a perennial SD plant. BMC Plant Biol..

[56] Iwata H (2012). The TFL1 homologue KSN is a regulator of continuous flowering in rose and strawberry. Plant J..

[57] Wang S (2014). An overview of the gene regulatory network controlling trichome development in the model plant, Arabidopsis. Regul. Cell Fate Determ. Plants.

[58] Abdelmaksoud EM, El-Refai SA, Mahmoud KW, Ragab ME (2020). Susceptibility of some new strawberry genotypes to infestation by western flower thrips, *Frankliniella occidentalis* (Pergande) (Thysanoptera: Thripidae) in the nursery. Ann. Agric. Sci..

