# Supplementary material for: TEMPRANILLO homologs in apple regulate flowering time in the woodland strawberry Fragaria vesca
Source: Sci Rep. 2023 Feb 3;13:1968. doi: 10.1038/s41598-023-29059-0 (PMC9898550; doi:10.1038/s41598-023-29059-0)
Supplement: Supplementary file 1 — Supplementary Information. [file 41598_2023_29059_MOESM1_ESM.docx]

# Supplementary Tables

**Supplementary Table 1** Primers used for PCR and quantitative Real-time PCR analyses

| Primer | Forward | Reverse | Tm | Size |
| --- | --- | --- | --- | --- |
| *FvMSI1* | TCTCCACACCTTTGATTGCCA | ACACCATCAGTCTCCTGCCAAG | 58 | 341 |
| *MdTEM‎1‎‏* | CTCTAAGTCCGAAATCGTCGACATGC | GGCTTCTTGCCAAACTCTAATTCCA | 58 | 729 |
| *MdTEM‎‎2* | GATTGGAAGACCAGGATGAGTGT | GGCTTCTTGCTAAACTCTAATTTCA | 58 | 242 |
| *tNOS* | GAATCCTGTTGCCGGTCTTG | TTATCCTAGTTTGCGCGCTA | 56 | 180 |
| *RNAi*-*MdTEM* | CTCTAAGTCCGAAATCGTCGACATGC | TGTTGTCCGTACCGAACAGGCC | 58 | 137 |
| *NPTII* | CTCGACGTTGTCACTGAAGCGGGAAG | AAAGCACGAGGAAGCGGTCAGCCCAT | 56 | 489 |
| *FvMSI1*-q | TCTCCACACCTTTGATTGCCA | ACACCATCAGTCTCCTGCCAAG | 60 | 107 |
| *FvFT1*-q | TGAGCTCAAACCTTCCCAAG | CAATCTCTTGGCCGAAAACT | 60 | 194 |
| *FvTEM‏-*q | ATGGACATAACTAGCAGCACAACAG | GAGCTTGTTGTGGTTGTGTTGGGTG | 60 | 105 |
| *FvGA3ox1-*q | CCTCACAATCATCCACCAATCC | CGCCAATGTTGATCACCAA | 60 | 110 |
| *FvGA3ox2‎-*q *‏* | GTGGCACGAGGGGTTTACTATCATGG | CCAGAGCACTGACTCTTAAGCCAACTCA | 58 | 204 |
| *MdTEM‎1‎‏*-q | CTCTAAGTCCGAAATCGTCGACATGC | TGTTGTCCGTACCGAACAGGCC | 58 | 137 |
| *MdTEM‎‎2-*q | GATTGGAAGACCAGGATGAGTGT | GGCTTCTTGCTAAACTCTAATTTCA | 58 | 242 |
| *MdFT*-q | GGGCAAGAGATAGTGTGTTATGAAAGTCC | CCTCCCTTTGGCAGTTAAAATAGACGAC | 62 | 199 |
| *MdActin*-q | GTGAGGCTCTATTCCAACCATC | GGAACACAAATTGGGCAAGTAT | 61 | 297 |

**Supplementary Table 2** Transformation efficiency of wild strawberry based on the percentage of PCR-positive plants out of the total number of inoculated explants

| Construct | No. of Inoculated explants | No. of PCR positive plants | Transformation efficiency |
| --- | --- | --- | --- |
| 35S::*MdTEM1* | 140 | 33 | 23.57 |
| 35S::*MdTEM2* | 164 | 28 | 17.07 |
| RNAi-*tem* | 120 | 39 | 32.5 |

**Supplementary Table 3** ANOVA of morphological flowering traits in wild strawberry

|  |  | Mean Square | | |
| --- | --- | --- | --- | --- |
| S.O.V | df | No. of leaves before flowering | No. of days before flowering | Trichome per mm^2^ |
| Treatment | 3 | 86.48^**^ | 300.54^**^ | 447.37^**^ |
| Error | 25 | 4.02 | 8.96 | 10.24 |
| C.V |  | 17.32 | 9.95 | 11.18 |
| ^ns^ no significant, *and ** significant at 5% and 1% probability level, respectively. | | | | |

# Supplementary Figs


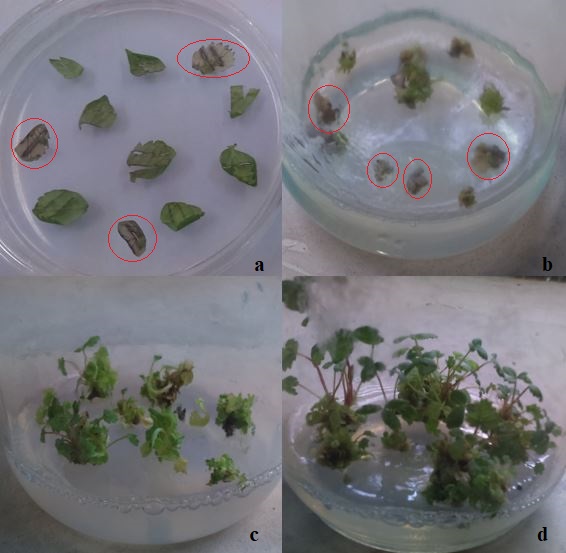


**Supplementary Fig. 1** Selection and regeneration of putative transgenic lines of wild strawberry. a and b) Untransformed explants became necrotic (red), c and d) regeneration and proliferation of putative transformed explants.


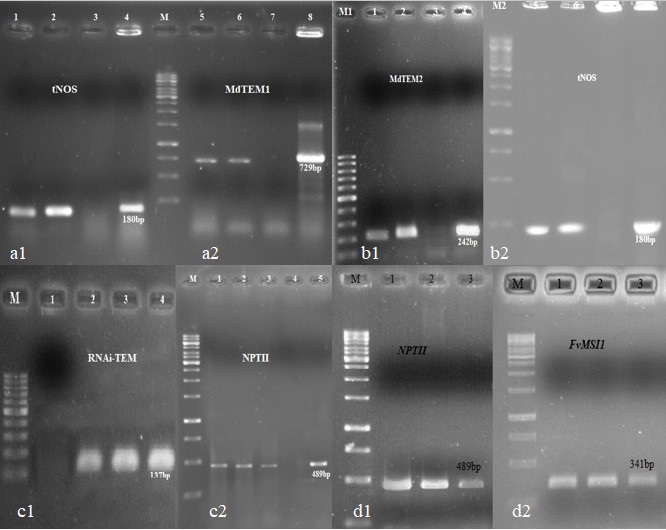


**Supplementary Fig. 2** Wild strawberry plant transformation confirmation by PCR using tNOS, MdTEM1 and MdTEM2 primers for overexpression lines, and RNAi-tem and NPTII primers for RNAi silencing lines. a1 and a2: *35S::MdTEM1* (1 and 2: *35S::MdTEM‎1* lines, 3: control plant, 4: plasmid control); b1 and b2: *35S::MdTEM‎2* (1 and 2: *35S::MdTEM‎*2 lines, 3: control plant, 4: plasmid control); c1 and c2: RNAi-*MdTEM* (2,3 and 4 (c1) and 1,2,3 (c2): three RNAi-*MdTEM* lines); d1 and d2: RT-PCR of RNAi-*MdTEM* using *NPTII* and *FvMSI1* primers.

**Supplementary Fig. 3** Early flowering in a) *RNAi*-tem silencing lines under inductive conditions; b) WT (16/8h light/dark photoperiod and 25°C) in growth chamber after 4.5 weeks and c,d) *RNAi*-tem silencing lines under *in-vitro* conditions (16/8h light/dark photoperiod and 25°C) after 4 weeks. Arrows are showing the flowers.


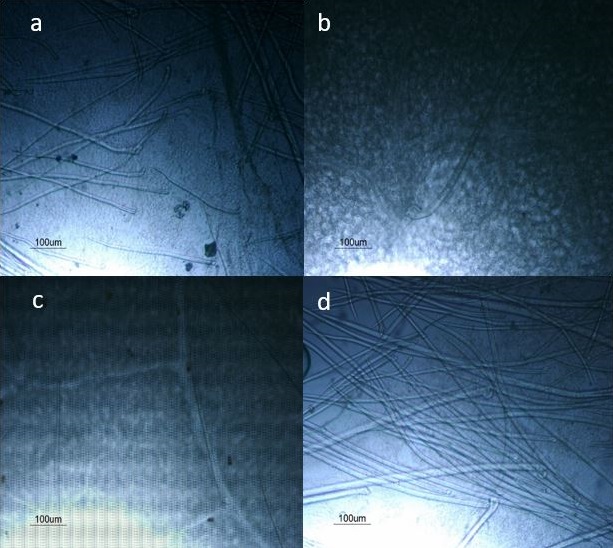


**Supplementary Fig. 4** Distribution pattern and density of trichomes on the abaxial side of *Fragaria vesca* transgenic lines leaves. a) Control b) 35S::*MdTEM‎1‎‏* c) 35S::*MdTEM‎‎2* d) RNAi-*TEM.*
